# Association between Neutrophil-to-Lymphocyte Ratio and Physical Function in Older Adults: A Community-Based Cross-Sectional Study in Japan

**DOI:** 10.3390/ijerph19158996

**Published:** 2022-07-24

**Authors:** Yuko Yoshida, Hajime Iwasa, Hunkyung Kim, Takao Suzuki

**Affiliations:** 1 Tokyo Metropolitan Institute of Gerontology, Tokyo 173-0015, Japan; hajimei@fmu.ac.jp (H.I.); kimhk@tmig.or.jp (H.K.); anant501@obirin.ac.jp (T.S.); 2 Department of Public Health, School of Medicine, Fukushima Medical University, Fukushima 960-1295, Japan; 3 Institute of Gerontology, J. F. Oberlin University, Tokyo 194-0294, Japan; 4 National Center for Geriatrics and Gerontology, Obu 474-8511, Japan

**Keywords:** physical function, neutrophil-to-lymphocyte ratio, community-dwelling older adults

## Abstract

Inflammatory responses contribute to physical decline in older adults. Clinical studies have shown that the neutrophil-to-lymphocyte ratio (NLR), a marker of inflammation, is associated with physical decline. However, its association with physical function in community-dwelling older people is still unclear. Hence, we used cross-sectional data to investigate the relationship between NLR and physical function in community-dwelling older adults. Specifically, we analyzed data corresponding to 818 individuals (336 men and 482 women) aged ≥ 75 years, all of whom participated in comprehensive health examinations, including face-to-face interviews, biochemical analyses, and physical function tests. Using these data, we performed multivariable logistic regression analysis to assess the relationship between NLR and physical function, adjusting for sex, age, education, alcohol consumption, smoking, instrumental activity of daily living, body mass index, chronic disease, physical activity, serum albumin level, and depressive mood. The results showed that a higher NLR was associated with a lower grip strength, lower knee extension strength, and slower walking speed. Importantly, the relationship between NLR and physical function was maintained after adjusting for the confounding factors. Thus, we showed a significant association between NLR and physical function, supporting the use of NLR as a marker of physical function in community-dwelling older adults.

## 1. Introduction

Physical function is an essential aspect of daily life that critically influences the quality of life. However, many older adults experience declined physical function, which is associated with a range of unhealthy conditions and risks, including falls [1], cognitive decline [2,3], hospitalization, death [4], and reduced well-being [5]. Thus, it is important for older adults to maintain physical function, especially to ensure independence.

Inflammation is known to affect physical function. Reportedly, a negative correlation exists between physical function and inflammatory markers, and higher C-reactive protein (CRP) [6,7] and interleukin-6 (IL-6) [6,8] levels are associated with lower physical function. The neutrophil-to-lymphocyte ratio (NLR) has also received considerable attention as an inflammatory indicator that reflects various health conditions. Notably, this systemic inflammatory response indicator [9], which is easily calculated based on the proportion of different white blood cell (WBC) fractions [10], can be obtained using a basic hemogram test. NLR, which is a widely used parameter in clinical practice, is calculated by simply dividing the total number of neutrophils by the total number of lymphocytes. It is also a useful indicator for screening inflammation in large populations. Furthermore, it is commonly measured in hospital-based studies and is associated with prognosis and frailty in patients with cancer [11,12] and with a higher probability of undergoing cardiac bypass surgery [13]. In addition, previous reports have suggested that it is associated with increased sarcopenia risk in hospitalized patients with cancer [14,15]. Additionally, Öztürk et al. [16] reported that increased NLR may be indicative of inflammation, which contributes to the development of sarcopenia in older adults. Thus, it can be used as a predictor of physical decline in older individuals.

Recently, it was suggested that NLR is an independent predictor of reduced walking speed in community-dwelling older people [17]. However, evidence that does not support this observation also exists. For example, a recent study by Tang et al. showed no significant association between inflammatory markers, including NLR, and sarcopenia [18]. This discrepancy highlights the need for further research to clarify suspected relationships and resolve any controversies. Thus, it is necessary to demonstrate the relationships between this inflammatory marker and physical function via more detailed investigations.

Even though NLR may be a key pathophysiological factor that either directly contributes to or mediates the mechanisms that lead to a decrease in physical function with age, the causal pathways responsible for NLR-mediated physical function impairment are not yet fully understood. To further clarify these relationships, researchers must control for several confounding variables, including physical activity, nutrition status, and depressive moods. In several studies, the involvement of these factors has been investigated. First, physical activity in the form of habitual exercise and training has been found to induce a decrease in NLR [19,20] and may exert an anti-inflammatory effect in individuals with chronic inflammation [21]. Additionally, participation in physical activity is known to have a positive effect on the maintenance of physical function in older adults [22,23].

Second, nutritional status must be considered with respect to a variety of aspects. For example, it has been demonstrated that undernutrition is associated with inflammation and reduced physical function. One study showed that older men with adequate protein intake have a more appropriate inflammatory status than their counterparts without adequate protein intake and that adequate protein intake may support anti-inflammatory processes and improve inflammatory status in cases of high inflammation [24]. Moreover, a high NLR is associated with a poor nutritional status in geriatric patients [25]. This is a critical concern, as inadequate nutrition is associated with poor physical function [26] and activity of daily living impairment [27].

Third, depressive status should be considered when assessing the relationship between inflammatory factors and physical function. Previous studies have shown that a high NLR is significantly associated with geriatric depression in women [28] and that it tends to be higher in patients with major depressive diseases [29]. Furthermore, a longitudinal study has also examined the relationship between depressive symptoms and disability and reported that depression is associated with increased functional impairment [30]. Similarly, a four-year prospective cohort study showed that individuals with depressive symptoms were at a higher risk of subsequent physical decline [31].

Thus, it is important to control each of these confounders when attempting to clarify the independent relationship between inflammatory factors and physical function. Additionally, most of the previous reports on NLR have been hospital-based and were focused on outpatients and inpatients with illness and functional disabilities. This implies that it is necessary to perform community-based studies targeting older people living independently in a community. Therefore, in this study, we aimed to evaluate the independent association between NLR and physical function in community-dwelling older adults.

## 2. Materials and Methods

### 2.1. Study Participants

In this study, we focused on cross-sectional data corresponding to 827 participants (341 men and 486 women) aged 75 years and above. This cross-sectional study was previously conducted as part of a project involving the comprehensive health examination of community-dwelling older people living in an urban area in Tokyo. Particularly, the health examination program was aimed at preventing functional decline and the need for long-term care in later life. This study is a 5-year follow-up of the project, and some of the data presented here have been previously described in other studies [32,33]. Of the 827 participants, 9 were excluded from the current analysis owing to missing biological data (***n*** = 6), physical function (*n* = 1), body mass index (BMI) (*n* = 1), and smoking habits (*n* = 1). Thus, the analyzed data corresponded to a total of 818 participants (336 men and 482 women).

### 2.2. Measurements

#### 2.2.1. NLR

Blood samples were collected from the antecubital vein without previous fasting when the participants were sedentary and in a sitting position. Thereafter, the collected blood samples were analyzed for WBCs (neutrophil and lymphocyte counts) and other hematological and biochemical indices. All the blood analyses were conducted at a certified commercial laboratory (Special Reference Laboratories, Tokyo, Japan). NLR was then calculated by dividing the total neutrophil count by the total lymphocyte count. Subsequently, the NLR data obtained were log-transformed and used for further analysis. Quartiles of the NLR values (Q1 to Q4) were defined separately by sex at the following cut-offs: ≤1.06, 1.07–1.50, 1.51–2.09, and >2.10 in men and ≤1.04, 1.05–1.46, 1.47–1.99, and >2.00 in women.

#### 2.2.2. Physical Function

Muscle strength and physical performance were assessed based on measurements for hand-grip strength, maximal knee extension power, the length of time the participants could stand on one leg, and usual walking speed. Hand-grip strength (kg) was assessed in the dominant arm using a Smedley’s hand dynamometer (Yagami, Tokyo, Japan), while maximal knee extension power (Nm) was measured using a hand-held dynamometer (MUSCLATOR GT-30, OG GIKEN, Tokyo, Japan) when participants were in the sitting position, with knee flexion at 90° degrees. The length of time the participants could stand on one leg (s) was assessed by having each participant stand on their preferred leg with their eyes open; the time (s) until they either lost their balance, or up to 60 s, was recorded. Finally, walking speed was measured while participants walked along a flat walkway (11 m). Specifically, over the 5 m distance between marks located at the 3 and 8 m positions, each participant was asked to walk at their usual pace. Walking speed (m/s) was then calculated by dividing the distance covered (m) by the time taken (s).

#### 2.2.3. Covariates

Face-to-face interviews were conducted to collect demographic information, including educational status, instrumental activity of daily living (IADL), drinking habits, smoking habits, regular physical activity, depressive mood, and chronic disease (hypertension, stroke, cardiovascular disease, hyperlipidemia, diabetes mellitus, and osteoporosis).

IADL was evaluated based on five items in the instrumental self-maintenance subsection of the 13-item Tokyo Metropolitan Institute of Gerontology Index of Competence [34]. Specifically, participants responded with either a “yes” or “no” to five items (e.g., “Can you use public transportation (bus or train) by yourself?”). Thereafter, each item was scored 1 for “yes” and 0 for “no”. The total scores were then tallied for each participant, with 5 points indicating “independence” and ≤4 points indicating “dependence”. Furthermore, chronic diseases were classified as binary, with at least one disease, and depressive mood was assessed via the Mini-International Neuropsychiatric Interview (M.I.N.I.) [35]. Thereafter, a binary classification was established, consisting of “with depressive mood” and “without depressive mood.” Nutritional status was assessed by measuring the levels of serum albumin, one of the parameters that reflect the nutritional status of older people [26,27], and was classified into binary values with a cut-off of 4.2 g/dL (normal) or more and 4.1 g/dL or less (lower nutrition). BMI was calculated as weight (kg) divided by the square of the height (m^2^). Thereafter, participants were categorized via binary classification, with a threshold of BMI ≥ 25 kg/m^2^.

### 2.3. Statistical Analyses

We examined the associations between NLR and physical function using the chi-square test, and, to compare differences in characteristics, we performed an analysis of variance. Furthermore, comparisons involving each physical function and NLR were realized by performing an analysis of covariance adjusted for sex and age. We also conducted a multivariable logistic regression analysis to clarify the associations between NLR and physical function, while controlling for sex, age, education, drinking status, smoking status, IADL, BMI, chronic disease, physical activity, serum albumin level, and depressive mood. Moreover, for multivariable logistic regression analysis, four items corresponding to each physical function were classified into binary values. First, the physical function values were classified into three groups (33rd percentiles) by sex and thereafter further categorized as “weak” and “other” dichotomous values. The lowest tertile was defined as “weak” with the following cut-off values for men and women, respectively: grip strength, <25 kg and <15 kg; knee muscle strength, <71.9 Nm and <47.3 Nm: one-legged standing time, <16.0 s and <14.7 s; and walking speed, <1.11 m/sec and <1.09 m/sec, respectively.

All the statistical analyses were performed using the SPSS software v27.0 for Windows (IBM Corp., Armonk, NY, USA). A two-tailed *p*-value < 0.05 was considered statistically significant.

## 3. Results

Table 1 lists the demographic characteristics of the participants. Of all the participants (mean age = 80.1 ± 3.7 years), 58.9% were female and 7.7% were IADL dependent. Furthermore, the mean NLR of all the participants was 1.67 ± 0.89.

Table 2 lists these characteristics according to the NLR level quartiles. As shown in the table, there were intergroup differences with respect to age (*p* = 0.037), physical activity (*p* < 0.001), and serum albumin level (*p* = 0.001). Notably, higher NLRs were found to be associated with an older age, lesser exercise, and lower serum albumin levels.

**Figure 1:**
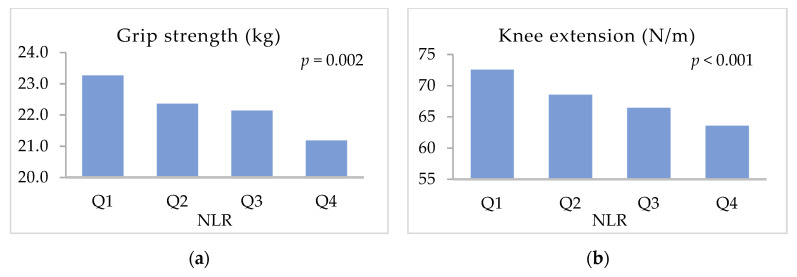

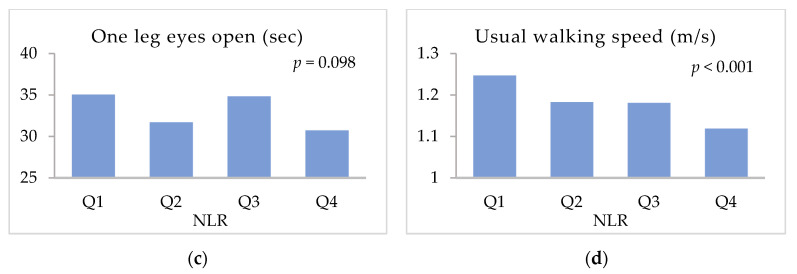
Variation of the adjusted means of physical functions with NLR level. (**a**) Grip strength; (**b**) knee extension; (**c**) standing on one leg with eyes open; and (**d**) usual walking speed. Results are based on the analysis of covariance adjusted for sex and age.

Figure 1 shows the physical function values (adjusted for sex and age) of the participants according to the NLR level quartiles. From this figure, it is evident that the different NLR quartiles showed differences in grip strength (*p* < 0.01), knee extension strength (*p* < 0.001), usual walking speed (*p* < 0.001), and maximal walking speed (*p* < 0.001). Furthermore, NLR and physical function were inversely related. However, no differences were observed between the NLR quartiles with respect to the time spent standing on one leg.

Table 3 shows the results of the multivariable logistic regression analysis for associations between NLR and physical function. NLR was negatively associated with physical function with respect to hand-grip strength (adjusted odds ratio [aOR] = 1.779, 95% confidence interval [CI] = 1.068–2.963 for Q4), knee extension (aOR = 1.800, 95 CI = 1.102–2.942 and aOR = 1.907, 95 CI = 1.164–3.122 for Q3 and Q4, respectively, *p* for trend = 0.049), and usual walking speed (aOR = 2.254, 95% CI = 1.375–3.695, aOR = 1.739, 95% CI = 1.048–2.884, and aOR = 2.375, 95% CI = 1.445–3.903 for Q2, Q3, and Q4, respectively, *p* for trend = 0.003) after adjusting for sex, age, education, drinking status, smoking status, IADL, BMI, chronic disease, physical activity, serum albumin level, and depressive mood. However, there were no significant associations between the NLR level quartiles and one-leg standing time.

## 4. Discussion

In this study, we investigated the relationship between NLR and physical function in community-dwelling older adults. Based on relevant measurements, we observed that NLR was associated with muscle strength in the upper and lower limbs and gait function, but it was not associated with balance capacity. Moreover, these associations remained after adjusting for confounding factors. Furthermore, our findings suggested that a higher NLR is associated with poorer physical function. Importantly, most relevant studies have been conducted in hospital settings, meaning that, generally, there is a lack of evidence in this regard in the context of community-dwelling individuals. Thus, our results provide a novel contribution to the literature and can help to resolve persistent controversies owing to differing results.

In previous studies, a negative correlation has been observed between physical function and inflammatory markers, with higher levels of CRP [6,7] and IL-6 [6,8] associated with poorer physical function. Meanwhile, less is known regarding the relationship between NLR and physical function in older adults.

NLR is a systemic inflammatory response index [9] which is thought to reflect the balance between innate (neutrophils) and adaptive (lymphocytes) immune responses. Moreover, previous studies have reported that NLR is associated with increased sarcopenia risk in hospitalized patients with cancer [14,15]. Similarly, a previous study on the association between sarcopenia and NLR revealed a higher NLR for patients in the sarcopenia group than that for those in the non-sarcopenia group, suggesting that an increased NLR indicates that inflammation plays an important role in the development of sarcopenia in older adults [16].

Additionally, there is evidence for the existence of an association between WBC subtypes and physical function [36]. A recent study showed that NLR independently predicts slow gait speed in older adults [17]. Our results in this study are consistent with these findings.

Among the physical functions investigated in this study, we observed that NLR was associated with muscle strength and walking speed but that NLR is not associated with the time spent standing on one leg, which is often used as an indicator of balance capacity. Previous studies have suggested that inflammation affects gait function via several mechanisms, including energy supply, perception, musculoskeletal involvement, and central and peripheral nervous system function [17]. Furthermore, a high NLR indicates a higher number of neutrophils relative to lymphocytes, and, considering that neutrophils produce oxidative metabolites, cytokines, and free radicals and also damage various organs [37], a high NLR may also be associated with impaired muscle tissue function. In the present study, we showed the existence of an association between NLR and walking speed and muscle strength, but not balance capacity, suggesting that inflammation may more severely affect the musculoskeletal system than the sensory and nervous systems. These observations indicated that NLR does not affect balance capacity. Nevertheless, more detailed studies involving other inflammatory markers and cytokine interactions are needed to validate this assertion.

In this study, we were unable to establish causal pathways showing that NLR leads to physical function impairment. However, our results suggest that NLR may be a key pathophysiological factor that either directly contributes to or influences the intermediary mechanisms that result in decreased physical function with aging. Overall, our findings indicated that an elevated NLR is associated with impaired physical function, specifically including reduced muscle strength and limited walking function, both of which significantly impact the maintenance of daily life in older adults. In such demographics, it is important to detect declining physical function at an earlier time so that appropriate countermeasures can be quickly initiated. Additionally, the current findings suggested that NLR may be an effective indicator for these types of issues in community-dwelling older adults. It has also been reported in previous studies that physical activity [19,20] and dietary quality [24] are associated with NLR. Therefore, in the future, it would be necessary to develop interventions that promote lifestyle changes that reduce systemic inflammation.

This study had some limitations. First, we did not adjust for the administration of immunosuppressants. Although we did adjust for the number of diseases, we did not account for specific diseases in the model. In this respect, it is undeniable that certain diseases may increase or decrease inflammation status to varying degrees. As such, adjusting for these factors may weaken the determined association. Second, we could not determine causality for the identified associations. Given our cross-sectional study design, we could neither assess the direction of the correlation between NLR and physical function nor prospectively analyze changes in physical function. This points to the need for further investigations, including longitudinal analyses, which may clarify at least one of the mechanisms underlying the relationship between spiraling inflammation and functional disability. Thus, it will be necessary to test which measurements are the most effective. Third, multiple demographic and lifestyle factors are associated with NLR [38]. In this study, we considered several confounding factors, including age, educational level, alcohol consumption, smoking, IADL, number of diseases, physical activity, serum albumin level, and depressive mood. While the association between NLR and physical function remained significant even after adjusting for these factors, it is likely that we did not account for all the possible influencing factors. Finally, this study was conducted solely in a Japanese community context. Therefore, caution should be exercised when attempting to generalize the results to other countries, especially those with different genetic and cultural backgrounds. Nonetheless, our findings constitute a significant contribution to the literature and may be used as a foundation for further investigations.

## 5. Conclusions

This study showed a significant association between NLR and physical function based on cross-sectional data corresponding to community-dwelling older adults. Of particular note, this study suggested that NLR is a useful marker of physical function in older adults. This is important, as NLR is a widely used indicator that is inexpensive and easy to measure and can be applied to facilitate early detection and intervention with respect to physical decline.

## Figures and Tables

**Table 1:** Participant characteristics.

Characteristic	(*n* = 818)
Sex (women; %)	482 (58.9)
Age (mean ± SD)	80.1 ± 3.7
Education (higher; %)	612 (74.8)
Drinking habits (current; %)	335 (41.0)
Smoking habits (current; %)	62 (7.6)
IADL (dependence; %)	63 (7.7)
Body mass index (≥25; %)	141 (17.2)
Chronic disease (≥1; %)	552 (67.5)
Physical activity (active; %)	668 (81.7)
Serum albumin (≤4.1 g/dl; %)	288 (35.2)
Depressive mood (depressive; %)	27 (3.3)
NLR (mean ± SD)	1.67 ± 0.89

IADL, instrumental activity of daily living; NLR, neutrophil-to-lymphocyte ratio. Age and NLR are expressed as the mean ± standard deviation, while the remaining characteristics are expressed as n (%).

**Table 2:** Participant characteristics as a function of NLR level.

	NLR Level Quartiles				
	Q1	Q2	Q3	Q4	
	(*n* = 201)	(*n* = 214)	(*n* = 196)	(*n* = 207)	*p*-Value
Quartile values of NLR					
Men	≤1.06	1.07–1.50	1.51–2.09	>2.10	
Women	≤1.04	1.05–1.46	1.47–1.99	>2.00	
Sex (women; %)	118 (58.7)	129 (60.3)	112 (57.1)	123 (59.4)	0.931
Age (mean ± SD)	79.8 ± 3.4	79.7 ± 3.5	80.5 ± 4.0	80.6 ± 3.8	0.037
Education (higher; %)	154 (76.6)	156 (72.9)	141 (71.9)	161 (77.8)	0.459
Drinking habits (current; %)	82 (40.8)	93 (43.5)	89 (45.4)	71 (34.3)	0.114
Smoking habits (current; %)	16 (8.0)	15 (7.0)	15 (7.7)	16(7.7)	0.985
IADL (dependence; %)	13 (6.5)	11 (5.1)	16 (8.2)	23 (11.1)	0.119
Body mass index (≥25; %)	42 (20.9)	36 (16.8)	35 (17.9)	28 (13.5)	0.266
Chronic disease (≥1; %)	128 (63.7)	152 (70.0)	135 (68.9)	137 (66.2)	0.410
Physical activity (active; %)	169 (84.1)	187 (87.4)	163 (83.2)	149 (72)	<0.001
Serum albumin (≤4.1 g/dL; %)	52 (25.9)	70 (32.7)	76 (38.8)	90 (43.5)	0.001
Depressive mood (depressive; %)	5 (2.5)	5 (2.3)	10 (5.1)	7 (3.4)	0.386

IADL, instrumental activity of daily living; SD, standard deviation. Tested using the chi-square test and analysis of variance.

**Table 3:** Associations between NLR and physical functions.

	Grip Strength				Knee Extension				One-Leg Standing Time				Usual Walking Speed			
	(*n* = 762)				(*n* = 697)				(*n* = 817)				(*n* = 817)			
	OR	95% CI		*p*-Value	OR	95% CI		*p*-Value	OR	95% CI		*p*-Value	OR	95% CI		*p*-Value
Sex	0.526	0.355	0.780	0.001	0.933	0.642	1.356	0.717	1.139	0.766	1.693	0.521	0.877	0.604	1.273	0.490
Age	1.174	1.118	1.233	<0.001	1.131	1.079	1.186	<0.001	1.130	1.076	1.186	<0.001	1.204	1.148	1.262	<0.001
Education	0.599	0.405	0.886	0.010	0.621	0.425	0.906	0.013	0.623	0.423	0.918	0.017	0.586	0.404	0.852	0.005
Drinking habits	0.744	0.503	1.100	0.138	0.672	0.464	0.974	0.036	1.224	0.830	1.805	0.307	0.838	0.580	1.212	0.349
Smoking habits	1.040	0.523	2.066	0.911	1.337	0.698	2.559	0.381	1.145	0.579	2.263	0.697	1.454	0.771	2.741	0.248
Body mass index	0.869	0.531	1.422	0.577	0.855	0.539	1.356	0.506	2.654	1.734	4.063	<0.001	1.569	1.021	2.411	0.040
IADL	1.438	0.778	2.656	0.246	1.195	0.642	2.222	0.575	3.044	1.706	5.431	<0.001	3.159	1.728	5.775	<0.001
Chronic disease	1.134	0.771	1.667	0.523	1.152	0.799	1.662	0.448	1.595	1.072	2.373	0.021	1.530	1.058	2.210	0.024
Physical activity	0.627	0.403	0.977	0.039	0.849	0.545	1.321	0.468	0.510	0.334	0.781	0.002	0.334	0.221	0.505	<0.001
Serum albumin	1.244	0.862	1.797	0.244	1.783	1.259	2.526	0.001	2.004	1.394	2.881	<0.001	1.224	0.862	1.738	0.259
Depressive mood	0.882	0.312	2.491	0.812	1.580	0.607	4.112	0.348	0.923	0.338	2.520	0.876	1.670	0.655	4.255	0.283
NLR Q1	(ref)				(ref)				(ref)				(ref)			
Q2	1.204	0.712	2.038	0.489	1.506	0.918	2.470	0.105	1.465	0.895	2.400	0.129	2.254	1.375	3.695	0.001
Q3	1.224	0.725	2.069	0.449	1.800	1.102	2.942	0.019	0.755	0.444	1.285	0.300	1.739	1.048	2.884	0.032
Q4	1.779	1.068	2.963	0.027	1.907	1.164	3.122	0.010	1.067	0.642	1.772	0.803	2.375	1.445	3.903	<0.001
*p* for trend				0.143				0.049				0.080				0.003

aOR = adjusted odds ratio; CI = confidence interval; ref = reference.

## Data Availability

These data are not appropriate for public disclosure owing to ethical issues. If you are a researcher who is interested in an analysis using these data, please request access to the confidential data from the Ethics Committee of the Tokyo Metropolitan Institute of Gerontology.
